# Effects of Two Linguistically Proximal Varieties on the Spectral and Coarticulatory Properties of Fricatives: Evidence from Athenian Greek and Cypriot Greek

**DOI:** 10.3389/fpsyg.2017.01945

**Published:** 2017-11-13

**Authors:** Charalambos Themistocleous

**Affiliations:** Department of Philosophy, Linguistics and Theory of Science, Centre for Linguistic Theory and Studies in Probability, University of Gothenburg, Gothenburg, Sweden

**Keywords:** spectral variation, spectral moments, coarticulation, fricatives, consonants, speech production, Athenian Greek, Cypriot Greek

## Abstract

Several studies have explored the acoustic structure of fricatives, yet there has been very little acoustic research on the effects of dialects on the production of fricatives. This article investigates the effects of two linguistically proximal Modern Greek dialects, Athenian Greek and Cypriot Greek on the temporal, spectral, and coarticulatory properties of fricatives and aims to determine the acoustic properties that convey information about these two dialects. Productions of voiced and voiceless labiodental, dental, alveolar, palatal, and velar fricatives were extracted from a speaking task from typically speaking female adult speakers (25 Cypriot Greek and 20 Athenian Greek speakers). Measures were made of spectral properties, using a spectral moments analysis. The formants of the following vowel were measured and second degree polynomials of the formant contours were calculated. The findings showed that Athenian Greek and Cypriot Greek fricatives differ in all spectral properties across all places of articulation. Also, the co-articulatory effects of fricatives on following vowel were different depending on the dialect. Duration, spectral moments, and the starting frequencies of *F*1, *F*2, *F*3, and *F*4 contributed the most to the classification of dialect. These findings provide a solid evidence base for the manifestation of dialectal information in the acoustic structure of fricatives.

## 1. Introduction

During the last few decades, there has been a surge of interest on the acoustic properties of fricative consonants. Fricatives are sounds characterized by complex production patterns that result in different acoustic spectral shapes (Ladefoged and Maddieson, 1996; Iskarous et al., 2011). However, the effects of dialects on fricatives' acoustic productions are poorly understood (see for a discussion Thomas, 2013, p. 116). Earlier research determined how linguistic categories, such as the place of articulation and voicing shape the spectral properties of fricatives (e.g., Hughes and Halle, 1956; Nittrouer et al., 1989; Baum and McNutt, 1990; Ladefoged and Maddieson, 1996; Jongman et al., 2000; Fox and Nissen, 2005; Shadle, 2010; Iskarous et al., 2011; Koenig et al., 2013), yet most of these findings are based on acoustic evidence from a single language variety (e.g., for Korean fricatives see Cho et al., 2002, for English fricatives see Tabain, 1998; Jongman et al., 2000; Iskarous et al., 2011). Despite the fact that a number of earlier studies showed that social factors, such as gender and age (e.g., see Jongman et al., 2000; Fox and Nissen, 2005; Li et al., 2016), education, social identity, social networks (e.g., Baran, 2014) and the place of origin, urban vs. rural (Dubois and Horvath, 1998; Kochetov, 2006; Stuart-Smith, 2007; Mazzaro, 2011) have significant effects on fricatives, the effects of dialect on fricatives acoustic structure are understudied.

The purpose of this study is to determine the acoustic characteristics of fricative productions in two linguistically proximal varieties: Athenian Greek and Cypriot Greek and establish the sociophonetic effects of these two varieties on fricatives' production. By determining the acoustic patterns of fricatives that differ in the two varieties, the study aims to establish which aspects of fricative spectra convey sociophonetic information about the distinct lingualities of Athenian Greek and Cypriot Greek speakers. The central thesis of this paper is that cross-dialectal studies of fricative's acoustic structure can reveal patterns that designate speakers of different dialectal groups. The findings of this study can be important as they can unveil patterns of language variation and change, which often as Labov (1994, p. 78) suggests, “[a]t the outset, and through most of their development, they are completely below the level of social awareness. No one notices them or talks about them, and even phonetically trained observers may be quite unconscious of them for many years.” Notably, such effects can potentially unveil the cognitive processes that bidialectal speakers employ to elicit information about the dialect from fricative spectra.

Earlier studies on Greek point to impressionistic differences in the production of Athenian Greek and Cypriot Greek fricatives (e.g., see Newton, 1972a,b; Vagiakakos, 1973) and to differences in the fricative inventories of Athenian Greek and Cypriot Greek. Namely, unlike Athenian Greek, Cypriot Greek is characterized by quantity distinctions in its fricatives (geminates vs. singletons) (see Table 1)^1^ and also includes in its phonemic inventory fricatives articulated at the post-alveolar place of articulation (Newton, 1972a,b; Vagiakakos, 1973; Arvaniti, 2000; Tserdanelis and Arvaniti, 2001; Botinis et al., 2004; Payne and Eftychiou, 2006; Armosti, 2009; Christodoulou, 2015)^2^.

**Table 1:** Athenian Greek (AG) and Cypriot Greek (CG) fricative consonants.

		**Labiod**.		**Dental**		**Alveolar**		**Postalveolar**		**Palatal**		**Velar**	
AG		f	v	θ	ð	s	z			ç	ʝ	x	ɣ
CG	Singl.	f	v	θ	ð	s	z	ʃ	ʒ	ç	ʝ	x	ɣ
	Gemin.	f:	v:	θ:	ð:	s:			ʒ:	ç:	ʝ:	x:	ɣ:

Notably, only a handful of studies provides acoustic evidence on Athenian Greek and Cypriot Greek fricatives: Nirgianaki (2014) who provided acoustic evidence on the Athenian Greek fricatives, two earlier pilot studies of ours that report acoustic evidence for four fricatives of the Cypriot Greek and Athenian Greek fricatives, i.e., [f, θ, ç, x] (Aristodemou et al., 2015; Themistocleous et al., 2016), and Eftychiou (2008) who investigates vowel elision and within this context, she reports acoustic measurements for the Cypriot Greek [s]. So, this study will be the first to provide comparative data from Athenian Greek and Cypriot Greek fricatives and it will show their coarticulatory effects on the following vowels.

To understand the effects of dialects on fricatives, we provide evidence from three distinct studies: (i) an investigation of the spectral and temporal properties of fricatives, using spectral moments analysis and measurements of fricative duration; (ii) an investigation of the co-articulatory effects of fricatives on the following vowel formants, using polynomial models of vowel formants; and (iii) a classification model of the contribution of fricatives' spectral and temporal properties together with the effects of fricative-vowel coarticulation.

## 2. Study 1: spectral properties

Study 1 investigates the effects of dialect on the acoustic structure of fricatives. Fricative spectra are characterized by frication noise that can be distinguished from the aperiodic energy in a mid-high frequency range that extends throughout fricatives production. Also, the periodicity that occurs simultaneously with frication distinguishes fricatives into voiced and voiceless. Depending on their spectral properties, fricatives can be grouped into sibilants (e.g., [s, z, ʃ, ʒ]) and non-sibilants [f, v, θ, ð] (e.g., Hughes and Halle, 1956; Jongman et al., 2000; Shadle, 2010). The sibilants are produced when the air jet is forced to pass across the upper teeth. The non-sibilants consist of a more distributed noise, which is produced when the air-jet runs across an inclined obstacle, such as the hard or the soft palate. The labiodental fricatives are produced very close to the mouth opening and can be considered a third category, in terms of their spectra and articulators involved (Shadle, 2010).

A long established technique that attempts to provide an account of the local and global properties of fricative spectra is the spectral moments analysis. An advantage of using spectral moments is that this method can enable the probabilistic analysis of fricative spectra (see also Koenig et al., 2013). In our earlier research, we employed spectral moments to specify the effects of the place of articulation and stress on fricatives (Aristodemou et al., 2015; Themistocleous et al., 2016). In this study, we employ spectral moments to determine the effects of dialect on fricative spectra. In the following, we present the main effects observed from the three different studies employed in this research and then we discuss their main findings.

### 2.1. Methodology

The recordings of this study were conducted between 2011 and 2012 in Athens, which is the capital city of Greece and in Nicosia, which is the capital city of Cyprus, and it is part of larger program that aims to understand the effects of dialects on the acoustic structure of speech sounds (see also Themistocleous, 2016, 2017b).

#### 2.1.1. Speakers

Fricative sounds were produced by 20 female speakers of Athenian Greek and 25 female speakers of Cypriot Greek born and raised in Athens and Nicosia, respectively. The reason for selecting female speakers is that in this study we are not interested in exploring the effects of gender on speech production and also in this way we avoid normalization for gender with respect to fricative spectra and vowels. At the time of the recording, i.e., during the years 2011–2012, the speakers were between 19 and 29 years old (years; months; mean = 22;8). Sociolinguistically the speakers represented a young and educated population. Specifically, all speakers were university students, from middle-class families, and bilingual in Greek and English (as a second language). Note that Cypriot Greek speakers were familiar with Athenian Greek from their interactions with Athenian Greek speakers, the media, the formal education etc. By contrast, Athenian Greek speakers have much less familiarity with Cypriot Greek. The speakers had no speech or hearing disorders or previous history of neurological, cognitive, orostructural problems.

#### 2.1.2. Speech material

The speech materials consisted of CVCV words (see Table 2). Each word contained a labiodental ([f v]), dental ([θ ð]), alveolar ([s z]), palatal ([ç ʝ]), and velar ([ɣ x]) fricative in both stressed and unstressed position. Note that Cypriot Greek postalveolar consonants ([ʃ ʒ]) have been also recorded but they are not reported in this study, since there are no corresponding Athenian Greek consonants at the post-alveolar place of articulation. To allow for the production of both velar and the palatal fricatives the speech material included two vowel environments after the fricative consonant, namely the vowels /a/ and /i/. The keywords were embedded in a carrier phrase, that varied slightly so as to sound more natural to the speakers of each dialect. Specifically, the carrier phrase for Athenian Greek was /ˈipa *keyword* ˈpali/ (I told *keyword* again) and for the Cypriot Greek experiment the carrier phrase was /ˈipa *keyword* ˈpale/ (I told *keyword* again). Also we added filler words in the speech material to distract speakers from focusing on the keywords of the experiment. Since all contextual effects are kept constant in all cases, other coarticulatory or prosodic effects on fricative productions or on vowels measured are not expected.

**Table 2:** Experimental material.

**Stress**	**[f]**		**[v]**		**[θ]**		**[ð]**	
S	ˈfisa	saˈfi	ˈvisa	saˈvi	'θisa	sa'θi	'ðisa	sa'ði
U	fiˈsa	ˈsafi	viˈsa	ˈsavi	θiˈsa	ˈsaθi	ðiˈsa	ˈsaði
S	ˈfasa	ˈsafa	ˈvasa	saˈva	ˈθasa	saˈθa	ˈðasa	saˈða
U	faˈsa	saˈfa	vaˈsa	ˈsava	θaˈsa	ˈsaθa	ðaˈsa	ˈsaða
	**[s]**		**[z]**		**[ç x]**		**[ʝ ɣ]**	
S	ˈsisa	siˈsa	ˈzisa	saˈzi	ˈçisa	saˈçi	ˈʝisa	saˈʝi
U	siˈsa	ˈsisa	ziˈsa	ˈsazi	çiˈsa	ˈsaçi	ʝiˈsa	ˈsaʝi
S	ˈsasa	saˈsa	ˈzasa	saˈza	ˈxasa	saˈxa	ˈɣasa	saˈɣa
U	saˈsa	ˈsasa	zaˈsa	ˈsaza	xaˈsa	ˈsaxa	ɣaˈsa	ˈsaɣa

Overall, the speech material consisted of 5,760 fricative productions, namely, 1,920 productions for the six fricatives of Athenian Greek (i.e., 20 speakers × 6 fricatives × 2 repetitions × 2 word positions × 2 stress conditions × 2 vowels) and 2,400 productions for the eight fricatives that can precede both vowels in Cypriot Greek (i.e., 25 speakers × 6 fricatives × 2 repetitions × 2 word positions × 2 stress conditions × 2 vowels) and 1,440 productions for the four fricatives that precede either vowel /i/ or /a/ (i.e., 45 speakers × 4 fricatives × 2 repetitions × 2 word positions × 2 stress conditions × 1 vowel).

The Athenian Greek speakers were recorded in a recording studio in Athens and the Cypriot Greek speakers were recorded in a quiet room at the University of Cyprus. To avoid influence from the experimenter's speech variety on participants' productions (like code-switching from one variety to another, as it is often the case with Cypriot Greek speakers), the instructions were given to the Athenian Greek speakers by an Athenian Greek speaking assistant whereas the author, a Cypriot Greek speaker himself, provided the instructions to Cypriot Greek speakers. The instructions did not include information about the purposes of the experiment. The only information we provided included basic instructions about the experimental setting, such the appropriate distance from the microphone. Subjects read the sentences written in Greek orthography in random order. A Zoom H4n audio recorder was used for the recording and the voice was sampled at 44.1 kHz. Praat (Boersma and Weenink, 2016) was used for segmentation and acoustic analysis, spectral moments were calculated in Praat using a modified version of DiCanio (2013)'s script. The onsets and offsets of the frication noise were determined both in the waveform and spectrogram. Also, the offsets and onsets of the *F*1 and *F*2 facilitated the segmentation.

#### 2.1.3. Statistics

Fricative spectra are measured at multiple windows and then the probability distribution of these measurements is estimated with moments:

- *Center of gravity* is a measure of the mean energy concentration of fricatives.
- *Standard Deviation* is a measure of the deviation of spectral values from the center of gravity.
- *Skewness* is a measure of the shape of the spectral distribution; a positive skewness indicates a right-tailed distribution and a negative skewness indicates a left-tailed distribution.
- *Kurtosis* is a measure of the shape of the distribution and indicates how heavy the tails of the distribution are. When the distribution is flat, the kurtosis is negative and when the distribution forms a peak, then the kurtosis is positive.

We analyzed the middle 80% of the total duration of the fricative by excluding a 10% from each side. Then the first four spectral moments that correspond to the center of gravity, standard deviation, skewness, and kurtosis were calculated from the fricative spectra. A linear mixed effects analysis was conducted with the center of gravity, standard deviation, skewness, kurtosis, and duration as response variables. The dialect, place of articulation, voicing, and stress were employed in the model as fixed factors. Random intercepts for speakers and keywords were added in the models (for an account on linear mixed-models see Baayen, 2008; Bates et al., 2015). The duration was log-transformed where needed to improve the model—these cases are reported in the Results section.

### 2.2. Results

Athenian Greek and Cypriot Greek fricatives differed in all spectral properties across all places of articulation. The mean and the standard deviation of spectral moments are reported in Table 3. The linear mixed effects models for the center of gravity and standard deviation are shown in Table 4 and those for skewness and kurtosis are reported in Table 5.

**Table 3:** The mean and SD of duration (in ms), center of gravity (in Hz), standard deviation (in Hz), skewness, and kurtosis of Athenian Greek (AG) and Cypriot Greek (CG) fricatives articulated at Dental, Labiodental, Alveolar, Palatal, and Velar place of articulation.

				**Duration**		**CoG**		***SD***		**Skewness**		**Kurtosis**	
				***M***	***SD***	***M***	***SD***	***M***	***SD***	***M***	***SD***	***M***	***SD***
CG	Labiod.	V	S	67	23	2,192	2,478	2,789	1,958	8	7	134	219
AG	Labiod.	V	S	77	20	2,650	1,947	3,199	1,457	4	4	41	103
CG	Dental	V	S	68	17	1,205	1,040	2,066	1,541	11	9	252	514
AG	Dental	V	S	79	20	1,387	1,143	2,002	1,217	7	5	101	139
CG	Alveolar	V	S	106	29	8,462	2,644	3,349	1,240	−1	2	4	7
AG	Alveolar	V	S	86	17	5,718	1,594	2,605	722	0	1	3	6
CG	Palatal	V	S	92	35	2,970	2,718	2,987	1,455	3	3	25	35
AG	Palatal	V	S	93	20	2,176	1,342	2,512	984	3	3	25	45
CG	Velar	V	S	66	23	1,536	1,086	2,096	1,366	8	5	112	128
AG	Velar	V	S	78	22	1,219	453	1,269	539	7	4	90	146
CG	Labiod.	VL	S	103	24	7,422	3,637	4,484	1,279	1	3	9	42
AG	Labiod.	VL	S	100	21	6,390	1,758	4,577	770	1	1	0	3
CG	Dental	VL	S	107	27	6,983	3,675	4,443	1,217	1	2	7	28
AG	Dental	VL	S	89	19	6,818	1,956	4,360	760	1	1	0	2
CG	Alveolar	VL	S	121	37	10,104	1,258	2,370	630	−1	2	5	30
AG	Alveolar	VL	S	111	24	6,933	1,152	1,977	559	1	1	4	4
CG	Palatal	VL	S	109	26	6,891	2,027	3,636	730	1	1	2	7
AG	Palatal	VL	S	106	21	6,094	758	2,789	480	1	0	2	2
CG	Velar	VL	S	103	25	2,810	975	2,730	1,026	4	2	22	30
AG	Velar	VL	S	96	21	2,695	836	2,272	759	3	1	13	13
CG	Labiod.	V	U	55	16	1,559	1,656	2,114	1,535	10	7	178	226
AG	Labiod.	V	U	59	14	2,087	1,692	2,628	1,386	5	5	70	142
CG	Dental	V	U	55	16	1,055	665	1,640	1,199	12	8	268	387
AG	Dental	V	U	64	14	1,224	983	1,641	1,079	8	5	131	165
CG	Alveolar	V	U	87	25	8,036	2,863	3,414	1,176	−1	2	4	8
AG	Alveolar	V	U	72	17	5,192	1,709	2,726	737	0	1	4	14
CG	Palatal	V	U	73	26	1,554	1,094	2,364	1,206	6	5	64	109
AG	Palatal	V	U	62	28	2,727	1,976	2,542	961	3	2	14	21
CG	Velar	V	U	52	19	1,387	824	1,833	1,162	9	7	186	354
AG	Velar	V	U	70	18	1,104	331	1,109	453	8	3	112	104
CG	Labiod.	VL	U	98	24	7,459	3,522	4,483	1,230	1	2	6	28
AG	Labiod.	VL	U	92	19	6,130	1,708	4,548	765	1	1	1	3
CG	Dental	VL	U	90	23	6,157	3,581	4,156	1,475	2	4	18	79
AG	Dental	VL	U	85	21	6,751	1,607	4,435	751	1	1	0	1
CG	Alveolar	VL	U	102	26	9,990	1,295	2,481	651	−1	1	3	11
AG	Alveolar	VL	U	99	21	7,005	688	1,926	348	1	1	4	3
CG	Palatal	VL	U	108	23	6,909	1,710	3,602	668	1	1	1	5
AG	Palatal	VL	U	103	21	6,026	781	2,671	442	1	1	3	2
CG	Velar	VL	U	103	21	2,950	1,131	2,757	1,107	4	3	30	62
AG	Velar	VL	U	91	17	2,559	665	2,150	716	3	1	15	14

**Table 4:** Results from the linear mixed effects models for the effects of the effects of dialect [Athenian Greek (AG) and Cypriot Greek (CG)], place of articulation, voicing, and stress on duration, center of gravity, and standard deviation.

		**Estimate**	***SE***	***df***	***t* value**	**Pr (>|*t*|)**
Duration	Intercept	−2.60	0.06	88	−44.21	0.001
	Alveolar	0.33	0.10	43	3.24	0.01
	AG	0.12	0.05	87	2.61	0.05
	Voiceless	0.30	0.08	50	3.69	0.01
	Alveolar:AG	−0.30	0.03	6,589	−8.95	0.001
	Alveolar:Voiceless	−0.45	0.14	40	−3.21	0.01
	AG:Voiceless	−0.19	0.03	6,594	−5.73	0.001
	Alveolar:AG:Voiceless	0.34	0.04	5,545	8.24	0.001
	Palatal:AG:Voiceless	0.13	0.06	6,566	2.15	0.05
	Palatal:AG:Unstressed	−0.22	0.07	6,066	−3.36	0.01
	Velar:AG:Unstressed	0.14	0.07	6,074	2.06	0.05
	Palatal:AG:Voiceless:Unstressed	0.21	0.09	6,414	2.27	0.05
Center of gravity	Intercept	7.69	0.10	70	78.30	0.001
	Dental	−0.80	0.17	37	−4.64	0.001
	Alveolar	1.27	0.17	37	7.39	0.001
	AG	0.31	0.07	287	4.60	0.001
	Voiceless	1.00	0.14	46	6.99	0.001
	Unstressed	−0.23	0.09	1,508	−2.50	0.05
	Dental:AG	−0.20	0.07	6,602	−2.72	0.01
	Alveolar:AG	−0.69	0.07	6,601	−9.52	0.001
	Palatal:AG	−0.46	0.10	6,582	−4.67	0.001
	Velar:AG	−0.44	0.10	6,581	−4.41	0.001
	Dental:Voiceless	0.69	0.23	38	3.00	0.01
	Alveolar:Voiceless	−1.30	0.24	34	−5.41	0.001
	AG:Voiceless	−0.34	0.07	6,597	−4.78	0.001
	Voiceless:Unstressed	0.31	0.11	2,257	2.69	0.01
	Dental:AG:Voiceless	0.39	0.10	6,592	3.95	0.001
	Alveolar:AG:Voiceless	0.37	0.09	6,604	4.13	0.001
	Palatal:AG:Voiceless	0.42	0.13	6,572	3.26	0.01
	Velar:AG:Voiceless	0.44	0.13	6,573	3.33	0.01
	Palatal:AG:Unstressed	0.64	0.14	6,572	4.55	0.001
	Palatal:AG:Voiceless:Unstressed	−0.58	0.20	6,291	−2.93	0.01
*SD*	Intercept	7.64	0.05	99	139.38	0.001
	Dental	−0.24	0.07	41	−3.37	0.01
	Alveolar	0.42	0.07	41	6.03	0.001
	Palatal	0.27	0.12	35	2.30	0.05
	AG	0.17	0.06	160	2.68	0.01
	Voiceless	0.73	0.07	60	11.24	0.001
	Unstressed	−0.31	0.06	140	−5.00	0.001
	Dental:AG	−0.18	0.06	6,081	−2.87	0.01
	Alveolar:AG	−0.43	0.06	6,078	−6.81	0.001
	Palatal:AG	−0.38	0.09	6,526	−4.42	0.001
	Velar:AG	−0.63	0.09	6,530	−7.25	0.001
	Dental:Voiceless	0.25	0.10	49	2.58	0.05
	Alveolar:Voiceless	−0.95	0.09	37	−10.11	0.001
	Palatal:Voiceless	−0.44	0.14	40	−3.10	0.01
	Velar:Voiceless	−0.33	0.15	42	−2.26	0.05
	AG:Voiceless	−0.15	0.06	5,473	−2.51	0.05
	Alveolar:Unstressed	0.34	0.10	51	3.45	0.01
	Voiceless:Unstressed	0.32	0.08	225	3.86	0.001
	Alveolar:AG:Voiceless	0.19	0.08	6,172	2.43	0.05
	Velar:AG:Voiceless	0.39	0.11	6,567	3.41	0.01
	Dental:Voiceless:Unstressed	−0.27	0.13	99	−2.19	0.05
	Alveolar:Voiceless:Unstressed	−0.30	0.12	81	−2.58	0.05
	Dental:AG:Voiceless:Unstressed	0.28	0.13	1,856	2.09	0.05

**Table 5:** Results from the linear mixed effects models for the effects of the effects of dialect [Athenian Greek (AG) and Cypriot Greek (CG)], place of articulation, voicing, and stress on skewness, and kutosis.

		**Estimate**	***SE***	***df***	***t* value**	**Pr (>|*t*|)**
Skewness	Intercept	1.64	0.14	52	11.80	0.001
	Dental	0.47	0.17	37	2.78	0.01
	Alveolar	−2.62	0.21	82	−12.57	0.001
	AG	−0.74	0.16	156	−4.57	0.001
	Voiceless	−1.53	0.17	70	−8.81	0.001
	Dental:AG	0.32	0.16	4,371	2.01	0.05
	Alveolar:AG	1.05	0.22	4,686	4.79	0.001
	Palatal:AG	0.49	0.22	4,705	2.19	0.05
	Velar:AG	0.75	0.22	4,695	3.45	0.01
	Alveolar:Voiceless	1.62	0.28	89	5.79	0.001
	Velar:Voiceless	0.82	0.35	40	2.31	0.05
	Alveolar:Unstressed	0.68	0.30	107	2.27	0.05
	Voiceless:Unstressed	−0.56	0.23	185	−2.40	0.05
	Dental:AG:Voiceless	−0.63	0.24	3,278	−2.62	0.01
	Alveolar:AG:Unstressed	−1.03	0.31	4,504	−3.28	0.01
Kurtosis	Intercept	3.76	0.19	13	19.63	0.001
	Dental	0.45	0.17	88	2.62	0.05
	Alveolar	−2.46	0.19	118	−13.23	0.001
	Palatal	−1.24	0.29	96	−4.25	0.001
	AG	−1.36	0.24	138	−5.71	0.001
	Voiceless	−2.80	0.20	179	−14.31	0.001
	Unstressed	0.47	0.18	134	2.66	0.01
	Dental:AG	0.78	0.22	3,729	3.54	0.001
	Alveolar:AG	0.96	0.24	4,028	4.04	0.001
	Palatal:AG	0.90	0.32	4,845	2.83	0.01
	Velar:AG	1.48	0.30	4,758	4.93	0.001
	Alveolar:Voiceless	2.19	0.25	130	8.59	0.001
	Palatal:Voiceless	0.88	0.42	173	2.11	0.05
	Velar:Voiceless	1.00	0.37	107	2.71	0.01
	Alveolar:Unstressed	−0.64	0.27	129	−2.40	0.05
	Dental:AG:Voiceless	−1.32	0.35	2,818	−3.77	0.001
	Alveolar:AG:Voiceless	0.95	0.32	3,896	2.99	0.01

*Center of Gravity*. More specifically, *Cypriot Greek alveolar and velar fricatives* had higher center of gravity than the corresponding Athenian Greek fricatives. By contrast, *Athenian Greek dental fricatives* had higher center of gravity than Cypriot Greek fricatives. In the labiodental and palatal places of articulation, *Cypriot Greek voiceless fricatives* are produced with higher center of gravity than Athenian Greek voiceless fricatives whereas the voiced fricatives had higher center of gravity in Athenian Greek. The effects are the following:

1. *Cypriot Greek* > *Athenian Greek*:
  - [f]. Cypriot Greek: *M* = 7,440, *SD* = 3,375; Athenian Greek: *M* = 6,260, *SD* = 1,736.
  - Alveolar fricatives. Cypriot Greek [s] *M* = 10,064, *SD* = 1,271, [z] *M* = 8,249, *SD* = 2,759; Athenian Greek: [s] *M* = 6,968, *SD* = 954, [z] *M* = 5,453, *SD* = 1,670.
  - [ç]. Cypriot Greek: *M* = 6,900, *SD* = 1,871; Athenian Greek *M* = 6,060, *SD* = 767.
  - Velar fricatives. Cypriot Greek: [x] *M* = 2,879, *SD* = 1,053; [ɣ] *M* = 1,461, *SD* = 961, Athenian Greek: [x] *M* = 2,627, *SD* = 756, [ɣ] *M* = 1,162, *SD* = 399.
2. *Cypriot Greek* < *Athenian Greek*:
  - [v]. Cypriot Greek: *M* = 1,909, *SD* = 2,170; Athenian Greek: *M* = 2,366, *SD* = 1,841.
  - Dental fricatives. Cypriot Greek [θ]: *M* = 6,567, *SD* = 3,646) and Cypriot Greek [ð]: *M* = 1,133, *SD* = 881; Athenian Greek [θ]: *M* = 6,790, *SD* = 1,816, Athenian Greek [ð]: *M* = 1,306, *SD* = 1,067.
  - [ʝ] Cypriot Greek *M* = 2,253, *SD* = 2,170; Athenian Greek: *M* = 2,443, *SD* = 1,696.

**Figure 1:**
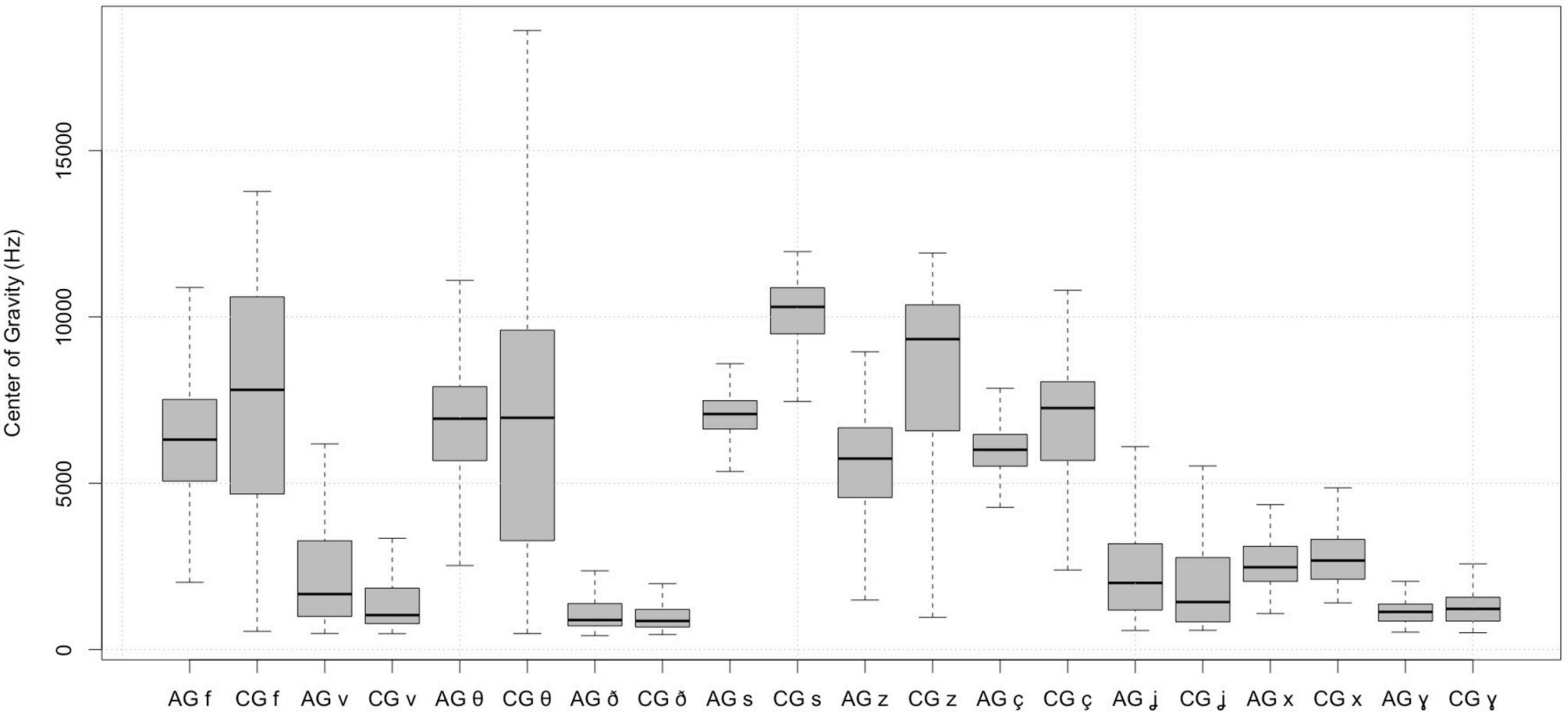
Spectral center of gravity of Athenian Greek (AG) and Cypriot Greek (CG) fricatives.

First, the dialect had an overall significant effect on the center of gravity (see also Figure 1). The interaction of dialect × place of articulation shows that the dental, alveolar, palatal and velar fricatives differ significantly in the two varieties. Also, the Athenian Greek and Cypriot [θ], [s], and [ç] differ significantly in their center of gravity. In addition to these effects, stress resulted in significantly different effects on the center of gravity of the Athenian Greek and Cypriot Greek *palatal* fricatives.

**Figure 2:**
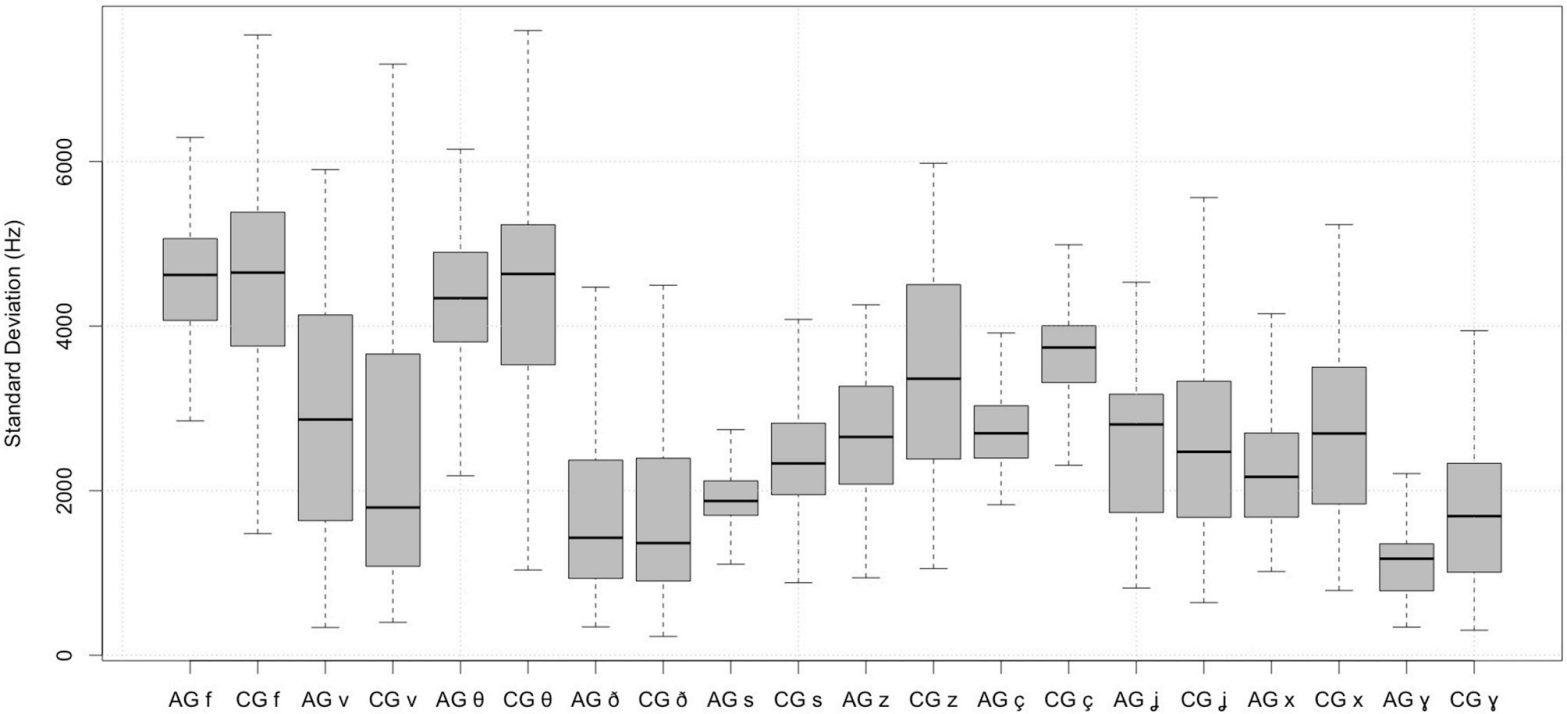
Spectral standard deviation of Athenian Greek (AG) and Cypriot Greek (CG) fricatives.

*Standard Deviation*. Dialect had significant effects on the spectral standard deviation of fricatives. Overall, Cypriot Greek fricatives are characterized by higher standard deviation than Athenian Greek fricatives (see also Figure 2). This is true for

- [v] (Cypriot Greek: *M* = 2,488, *SD* = 1,811, Athenian Greek: *M* = 2,911, *SD* = 1,447,
- [ð] (Cypriot Greek: *M* = 1,861, *SD* = 1,402, Athenian Greek: *M* = 1,822, *SD* = 1,162,
- the alveolars [s] (Cypriot Greek: *M* = 2,409, *SD* = 640, Athenian Greek: *M* = 1,952, *SD* = 468) and [z] (Cypriot Greek: *M* = 3,382, *SD* = 1,206, Athenian Greek: *M* = 2,666, *SD* = 730,
- the palatals [ç] (Cypriot Greek: *M* = 3,619, *SD* = 697, Athenian Greek: *M* = 2,731, *SD* = 463), [ʝ] (Cypriot Greek: *M* = 2,671, *SD* = 1,362, Athenian Greek: *M* = 2,527, *SD* = 969) and
- the velars [x] (Cypriot Greek: *M* = 2,743, *SD* = 1,062, Athenian Greek: *M* = 2,211, *SD* = 738) and [ɣ] (Cypriot Greek = *M* = 1,965, *SD* = 1,266, Athenian Greek: *M* = 1,189, *SD* = 502.

The results suggest that Cypriot Greek speakers produced all these fricatives with greater variation with respect to the center of gravity than Athenian Greek speakers. Only the Athenian Greek voiceless labiodental [f] (Cypriot Greek: *M* = 4,483, *SD* = 1,253, Athenian Greek: *M* = 4,563, *SD* = 766) and the dental [θ] (Cypriot Greek: *M* = 4,299, *SD* = 1,358, Athenian Greek: *M* = 4,391, *SD* = 756) had higher standard deviation than the corresponding Cypriot Greek fricative productions. Specifically, the two dialects had an overall effect on the spectral standard deviation, especially in dental, alveolar, palatal, and velar places of articulation. Also, there were significant differences in the standard deviation between the Athenian Greek and Cypriot Greek [s] and [x].

**Figure 3:**
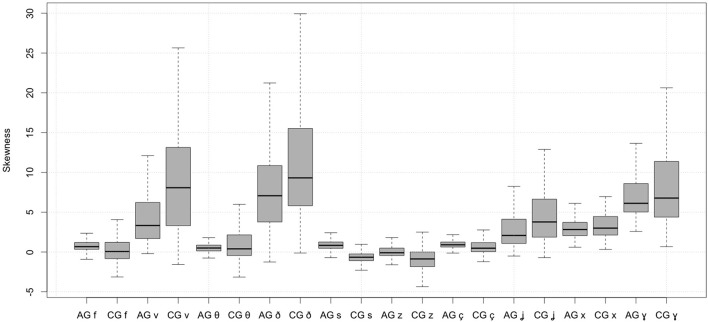
Spectral skewness of Athenian Greek (AG) and Cypriot Greek (CG) fricatives.

*Skewness*. The effects of skewness are shown in Figure 3. The boxplots in the figure represent the quantiles of skewness, namely the minimum value of skewness, the first quantile, the median, the third quantile, and the maximum skewness for each fricative. The upper and lower edge of the whiskers stand for the maximum and minimum value, respectively; the top and bottom of the box represent the third and first quantile and the black solid horizontal line in the middle of the box displays the median of the distribution. It is apparent from this figure that voiced fricatives differ from the voiceless ones in their skewness. Therefore it is not unexpected that voicing resulted in significant effects on skewness: voiced labiodental, palatal, and velar fricatives are characterized by relatively high skewness whereas alveolars and voiceless labiodental, palatal, and velar fricatives are characterized by relatively low skewness. Cypriot Greek alveolars display negative skewness whereas Athenian Greek alveolars are characterized by positive skewness. This issue will be discussed later in section 5. Overall, the dialect had an overall significant effect (see the results of the statistical model in Table 5). More specifically, there were significant differences between Athenian Greek and Cypriot Greek fricatives in the skewness of dental, alveolar, palatal, and velar fricatives. There were also significant effects of the place of articulation on skewness. This is evident for dentals and the alveolars. Also the dialect had a significant effect on the skewness of [θ]. Finally, dialect had significant effects on the stressed vs. unstressed [s] and [z].

**Figure 4:**
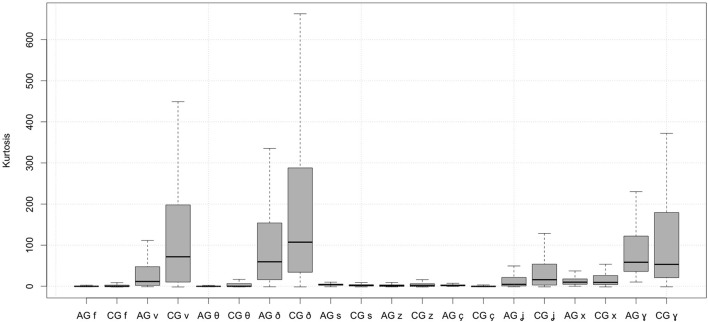
Spectral kurtosis of Athenian Greek (AG) and Cypriot Greek (CG) fricatives.

*Kurtosis*. The effects of Athenian Greek and Cypriot Greek fricatives on kurtosis are shown in Figure 4, which just like Figure 3, represents the quantiles of kurtosis using boxplots. The figure shows that voiced labiodental, dental, palatal, and velar fricatives have an extremely high kurtosis. By contrast, the kurtosis of voiceless fricatives and that of [z] is close to zero. Cypriot Greek fricatives associate with higher kurtosis than the corresponding Athenian Greek fricatives. These effects are more prominent in the voiced condition. Consequently, Athenian Greek and Cypriot Greek fricatives resulted in statistically significant effects on kurtosis (see the results of the statistical model in Table 5). Also, there were significant effects of the dialect on the kurtosis of dental, alveolar, palatal, and velar fricatives. Moreover, voiceless dental, voiceless alveolar, voiceless palatal, and voiceless velar Athenian Greek fricatives differed significantly from the corresponding Cypriot Greek fricatives.

#### 2.2.1. Temporal properties of fricatives

The statistical analysis shows significant effects of dialect on fricative duration. Overall, Cypriot Greek fricatives are on average longer (96 ms) than Athenian Greek fricatives (92 ms). What stands out in this analysis is the interactions of dialect × place of articulation, dialect × voicing, which showed significantly different effects for Athenian Greek & alveolar fricatives (alveolar fricatives are the longest fricatives) and Athenian Greek & voiceless fricatives on duration. The latter suggests that voiced and voiceless fricatives differ in their duration in Athenian Greek and Cyprio Greek.

There were also significant results from the interactions (1) place of articulation × dialect × voicing, (2) place of articulation × dialect × stress, and (3) place of articulation × dialect × voicing × stress. Specifically, the first interaction resulted in significantly different effects for the Athenian Greek voiceless alveolar ([s]) and palatal ([ç]) fricatives. The second resulted in significantly different effects for the Athenian Greek unstressed palatals ([ç, ʝ]) and velars ([x, ɣ]) and the third interaction resulted in significantly different effects for the unstressed Athenian Greek [ç]. Another factor that influences the duration of fricatives in both varieties is voicing. Specifically, voiceless fricatives are overall longer than the voiced ones.

To conclude, dialect affects fricative spectra systematically as in evident by the effects of dialect on fricatives' spectral moments (e.g., center of gravity, standard deviation, skewness, and kurtosis) and duration. The following section describes Study 2 of this work.

## 3. Study 2: fricative-vowel coarticulation

Earlier research has demonstrated that the coarticulatory effects of fricatives on a following vowel can provide information about fricatives' place of articulation and voicing (e.g., see Potter et al., 1947; Cooper et al., 1952; Stevens and House, 1956; Harris et al., 1958; Lehiste and Peterson, 1961; Öhman, 1966; Fant, 1969; de Manrique and Massone, 1981b; Kewley-Port, 1982; Beckman et al., 2009). However, the effects of dialect on fricative-vowel coarticulation received so far very little attention. Study 2 aims to provide evidence of the effects of dialect on fricative-vowel coarticulation. Specifically, it investigates the effects of Athenian Greek and Cypriot Greek fricatives on the polynomial coefficients of *F*1, *F*2, *F*3, and *F*4 formant contours. To this purpose, the formants were modeled using second degree polynomial models, which for the purposes of this study have a number of advantages: they represent the starting frequency of the formant, the shape of the overall formant contour, and they reduce the amount of measurements taken across the duration of the vowels into a small number of polynomial coefficients, which facilitates the statistical analysis (see for a discussion of this approach Themistocleous, 2017a). The innovative aspect of this study is that it explores for the first time the effects of dialect on fricative-vowel coarticulation and it is also the first study to investigate these effects in Greek dialects.

### 3.1. Methodology

We employed the same speech material as in Study 1; the specifics of the statistical analysis and the results are described in the following.

#### 3.1.1. Statistics

To model formant dynamics, we performed 13 measurements of *F*1, *F*2, *F*3, and *F*4 at 13 equidistant points starting from the 20–80% (included) (see also Jacewicz et al., 2011, p. 686). The measurements of *F*1, *F*2, *F*3, and *F*4 were fitted using a 2nd order polynomial fit. The second degree polynomial results into three coefficients:

- The *zeroth coefficient* (*a*_0_), which represents the starting frequency of the vowel formant;
- the *first order coefficient* (*a*_1_) and the *second order coefficient* (*a*_2_), which determine the shape formant contour.

**Figure 5:**
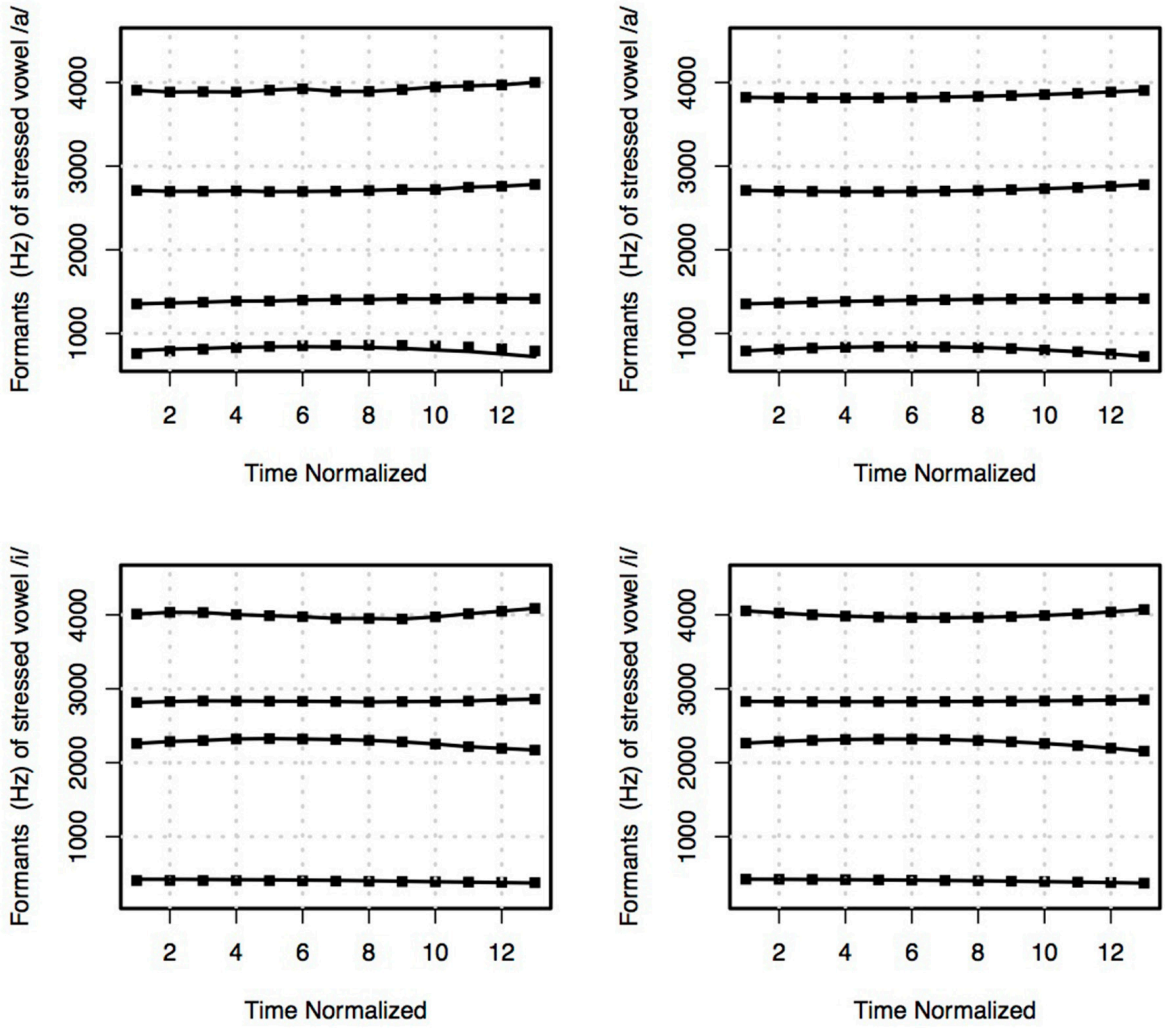
Means of F1−F4 (in Hz) of the actual vowel productions (Left panels) of the stressed vowels /a/ (Upper panel) and in /i/ (Lower panel) and the models of F1−F4 (in Hz) that resulted from the polynomial modeling.

The outputs of these models are smoothed representations of formant contours; an example is provided in Figure 5. Linear mixed effect models were employed to analyze formant dynamics, with the polynomial coefficients as response variables and the dialect, place of articulation, stress, voicing, and vowel as fixed factors. Keyword and speaker were employed as random effects, the resulting model is shown in Equation (1).

(1) $$\begin{array}{l} response\sim Dialect^{*}Placeofarticulation^{*}Stress^{*}Voicing\\ \;*Vowel+(1|Keyword)+(1|Speaker) \end{array}$$

### 3.2. Results

**Figure 6:**
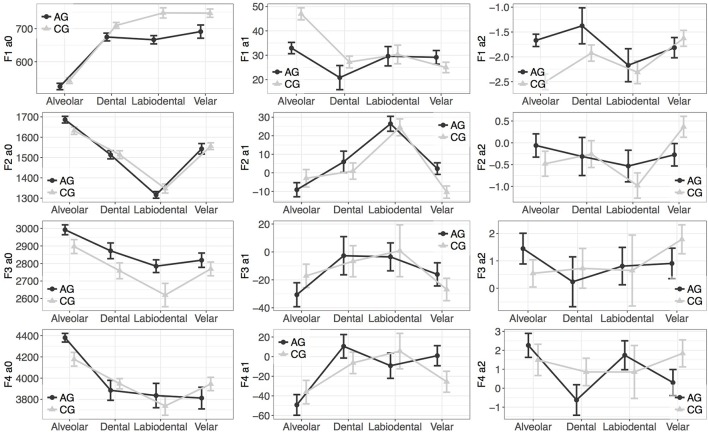
Means and SD of the polynomial coefficients of the vowel formants of /a/, preceded by the unstressed voiced alveolar, dental, labiodental, and velar fricatives.

**Figure 7:**
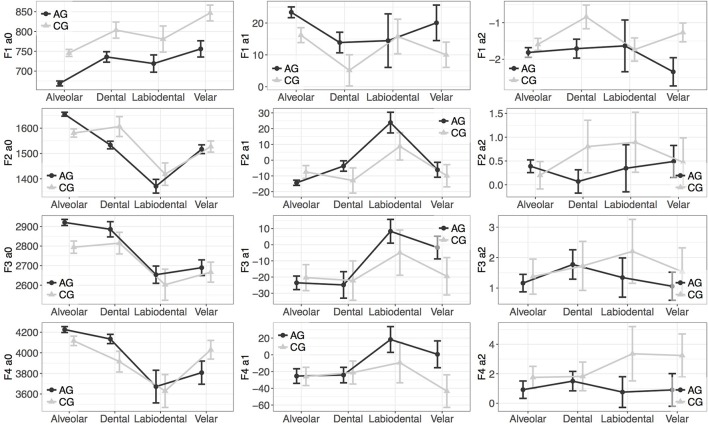
Means and SD of the polynomial coefficients of the vowel formants of /a/, preceded by the unstressed voiceless alveolar, dental, labiodental, and velar fricatives.

The means of the polynomial coefficients of *F*1 and *F*2 are shown in Table 6 and of *F*3 and *F*4 are shown in Table 7. The results of *F*1 and *F*2 are shown in Table 8 and those of *F*3 and *F*4 are shown in Table 8. Figures 6, 7 show an example of the specific interactions of place of articulation, stress, and variety on the coefficients of the stressed and unstressed vowel [a], respectively.

**Table 6:** Mean and *SD* of *a*_0_, *a*_1_, *a*_2_ polynomial coefficients of the formant frequencies *F*1 and *F*2 of vowels /a/ and /i/ as a function of fricative consonant and dialect [Athenian Greek (AG) and Cypriot Greek (CG)].

			**F1**						**F2**					
			***a***_**0**_		***a***_**1**_		***a***_**2**_		***a***_**0**_		***a***_**1**_		***a***_**2**_	
			***M***	***SD***	***M***	***SD***	***M***	***SD***	***M***	***SD***	***M***	***SD***	***M***	***SD***
ð	CG	a	735.26	176.41	28.90	29.93	−2.01	1.96	1, 525.56	215.18	−0.23	46.66	0.00	3.60
f	CG	a	812.63	124.58	19.75	27.99	−1.81	1.91	1, 401.23	182.34	10.53	39.04	−0.34	2.60
ɣ	CG	a	804.34	163.59	24.85	37.86	−1.94	2.54	1, 616.46	311.74	−16.01	65.98	1.93	4.55
s	CG	a	814.59	114.17	24.93	29.20	−2.03	2.07	1, 587.43	233.89	0.52	62.34	−0.36	4.12
v	CG	a	775.33	125.35	32.46	28.55	−2.39	1.98	1, 369.48	234.52	24.01	61.41	−1.12	4.26
x	CG	a	879.89	132.44	10.51	31.42	−1.23	2.13	1, 531.78	251.88	−3.58	63.54	0.10	4.07
z	CG	a	601.56	152.52	48.13	29.12	−2.74	1.87	1, 613.01	159.49	−3.76	50.45	−0.14	3.73
θ	CG	a	832.01	132.22	9.54	33.82	−1.10	2.65	1, 566.99	152.98	−1.85	56.11	0.09	4.11
ð	AG	a	721.60	115.99	26.93	33.27	−1.66	2.66	1, 513.34	134.82	10.54	34.23	−0.64	2.81
f	AG	a	755.62	86.51	23.10	23.93	−2.10	1.84	1, 349.06	119.44	15.23	30.73	−0.29	2.46
ɣ	AG	a	708.58	104.60	33.93	30.44	−2.07	2.30	1, 548.40	159.06	−0.47	38.72	0.72	2.82
s	AG	a	725.92	109.20	35.56	32.49	−2.33	2.36	1, 647.05	158.27	−18.73	40.45	0.78	2.92
v	AG	a	707.44	92.01	32.63	24.53	−2.15	1.83	1, 329.65	147.04	21.32	38.87	−0.17	2.73
x	AG	a	809.41	95.25	21.43	25.09	−2.35	1.41	1, 508.41	148.40	−3.37	29.16	0.35	2.06
z	AG	a	572.68	147.03	40.53	36.46	−2.10	2.62	1, 712.28	132.31	−16.04	36.23	0.57	2.82
θ	AG	a	756.51	131.46	22.97	23.45	−2.10	1.57	1, 524.35	119.08	−6.50	28.34	0.40	1.89
ç	CG	i	415.61	166.49	3.27	41.55	−0.30	3.40	2, 620.06	338.37	−0.24	134.41	−1.58	8.91
ð	CG	i	451.25	84.32	−1.51	33.18	−0.12	2.32	2, 270.99	458.01	46.39	105.77	−3.00	6.68
f	CG	i	413.49	380.48	−2.70	105.37	0.10	7.84	2, 528.54	399.17	−12.53	96.21	−0.56	6.66
ʝ	CG	i	377.83	142.65	7.25	42.09	−0.36	3.51	2, 565.12	420.07	4.01	89.15	−0.80	5.71
s	CG	i	409.00	91.14	4.17	19.77	−0.43	1.49	2, 489.02	333.91	9.21	107.04	−1.65	8.34
v	CG	i	417.54	50.10	2.81	11.19	−0.29	0.79	2, 210.65	300.27	76.45	93.12	−5.08	6.20
z	CG	i	395.28	200.82	3.66	15.75	−0.24	1.09	2, 230.58	452.61	27.37	123.24	−1.39	8.25
θ	CG	i	462.69	93.05	−2.23	12.69	0.06	0.91	2, 479.16	498.24	11.60	119.36	−1.32	7.77
ç	AG	i	357.97	62.03	7.05	24.89	−0.48	2.45	2, 495.79	235.85	1.37	71.63	−1.30	4.93
ð	AG	i	426.00	58.31	0.53	11.57	−0.26	0.74	2, 109.28	258.39	51.81	88.25	−2.37	5.97
f	AG	i	416.91	63.12	−2.20	10.20	−0.09	0.77	2, 227.12	314.85	33.62	106.42	−2.71	7.24
ʝ	AG	i	337.34	71.05	7.55	12.94	−0.34	0.71	2, 603.05	325.23	−10.49	91.48	0.21	6.22
s	AG	i	408.95	77.29	−1.07	23.60	−0.11	1.84	2, 225.70	219.94	28.07	64.14	−1.72	4.91
v	AG	i	415.12	60.15	3.11	11.83	−0.47	0.73	2, 074.96	235.40	60.85	69.04	−2.94	4.95
z	AG	i	373.93	60.78	6.40	14.82	−0.45	1.14	2, 105.97	313.05	20.39	78.55	−0.49	5.95
θ	AG	i	420.44	50.31	0.14	8.32	−0.36	0.60	2, 259.77	372.14	33.75	96.14	−2.74	6.31

**Table 7:** Mean and SD of *a*_0_, *a*_1_, *a*_2_ polynomial coefficients of the formant frequencies *F*3, and *F*4 of vowels /a/ and /i/ as a function of fricatiVe consonant and dialect [Athenian Greek (AG) and Cypriot Greek (CG)].

			**F3**						**F4**					
			***a***_**0**_		***a***_**1**_		***a***_**2**_		***a***_**0**_		***a***_**1**_		***a***_**2**_	
			***M***	***SD***	***M***	***SD***	***M***	***SD***	***M***	***SD***	***M***	***SD***	***M***	***SD***
ð	CG	a	2, 774.22	475.41	−11.46	102.81	1.03	7.90	3, 905.00	858.62	−6.44	141.46	0.80	10.74
f	CG	a	2, 595.89	539.51	−5.43	133.94	0.85	9.31	3, 723.81	714.02	−12.88	152.17	1.64	11.32
ɣ	CG	a	2, 836.10	562.11	−68.32	115.12	5.24	7.38	4, 036.25	911.61	−69.75	153.89	5.86	10.60
s	CG	a	2, 706.70	574.57	−10.70	143.49	0.88	9.41	4, 010.68	741.44	−46.34	145.37	3.04	9.81
v	CG	a	2, 624.01	540.68	3.74	141.80	0.19	9.50	3, 747.70	735.82	14.83	192.45	−0.32	13.14
x	CG	a	2, 637.52	540.11	−3.27	137.34	0.32	8.88	4, 002.61	774.17	−30.65	188.89	1.89	12.05
z	CG	a	2, 829.60	421.96	−22.05	112.39	1.33	7.99	4, 134.47	804.42	−43.45	169.42	2.54	12.02
θ	CG	a	2, 742.93	440.80	−13.43	116.48	1.33	7.81	3, 886.58	757.58	−22.35	187.32	2.00	13.23
ð	AG	a	2, 797.88	281.63	8.34	85.25	−0.53	6.15	3, 853.63	739.57	2.90	104.34	0.06	7.50
f	AG	a	2, 703.24	294.30	−5.85	66.38	1.21	4.88	3, 789.89	748.22	−1.34	88.91	0.99	5.90
ɣ	AG	a	2, 779.39	426.28	−37.56	101.49	2.75	6.46	3, 874.37	467.10	−35.80	103.87	2.71	7.72
s	AG	a	2, 843.61	441.42	−38.95	111.89	2.76	6.99	4, 029.19	861.63	−37.12	122.15	2.53	7.91
v	AG	a	2, 750.19	381.15	−16.64	106.70	1.89	7.32	3, 843.26	713.77	−16.45	160.21	1.99	11.47
x	AG	a	2, 686.71	293.36	−8.74	96.60	1.53	6.27	3, 877.85	420.11	−13.34	118.68	1.57	7.29
z	AG	a	3, 007.44	301.24	−51.79	70.88	2.99	5.54	4, 383.61	831.56	−74.22	138.24	4.07	9.89
θ	AG	a	2, 836.62	243.75	−28.07	88.98	2.37	6.14	4, 053.78	939.04	−26.57	126.69	1.90	8.65
ç	CG	i	3, 363.96	285.30	−20.94	75.36	−0.19	5.27	4, 060.68	999.83	−12.50	116.33	0.68	7.66
ð	CG	i	2, 938.27	267.77	19.64	64.44	−1.41	4.23	3, 978.51	678.79	0.35	107.67	0.29	7.36
f	CG	i	3, 004.53	320.47	3.86	77.20	−0.58	5.75	3, 884.16	1085.45	9.95	127.57	−0.37	8.49
ʝ	CG	i	3, 447.62	298.87	−39.31	54.97	1.26	3.76	4, 251.87	897.64	−36.48	115.06	3.00	7.82
s	CG	i	3, 083.85	275.99	−2.65	64.51	−0.14	5.05	4, 154.57	904.33	−8.82	101.17	0.36	6.90
v	CG	i	2, 832.56	261.75	44.74	59.52	−2.64	3.80	3, 918.82	641.29	22.14	117.22	−0.79	7.67
z	CG	i	3, 016.22	314.42	−3.47	76.30	0.05	5.56	4, 306.86	942.18	−27.44	89.78	1.32	6.21
θ	CG	i	3, 097.39	340.06	−0.42	83.26	−0.45	5.62	4, 002.70	586.77	−8.73	105.11	0.78	7.90
ç	AG	i	3, 233.71	184.38	−43.52	48.65	2.07	3.62	4, 037.68	474.65	−10.09	147.30	1.36	9.77
ð	AG	i	2, 886.32	208.81	19.02	45.68	−0.80	3.14	4, 016.28	427.79	8.15	125.94	0.31	9.21
f	AG	i	2, 834.87	209.14	0.09	53.96	0.35	3.62	4, 102.05	611.47	−24.64	136.22	2.16	8.83
ʝ	AG	i	3, 478.42	227.53	−48.26	59.10	1.52	3.96	4, 264.40	788.68	−37.35	151.67	2.41	10.13
s	AG	i	2, 948.50	177.63	−1.98	50.82	0.19	3.65	4, 224.17	521.44	−14.79	110.20	1.19	7.23
v	AG	i	2, 767.86	270.30	20.78	60.45	−0.24	4.28	4, 017.95	387.75	−11.79	116.15	2.40	8.38
z	AG	i	3, 038.69	261.89	−21.76	70.70	1.31	5.34	4, 341.79	615.11	−18.95	102.16	1.05	7.34
θ	AG	i	2, 909.32	252.20	−1.31	65.20	0.04	4.15	4, 173.50	639.70	−24.60	91.16	2.12	5.52

**Table 8:** Effects of dialect [Athenian Greek (AG) and Cypriot Greek (CG)], place of articulation, voicing, stress, and vowel on the three polynomial coefficients of *F*1 and *F*2.

		**Estimate**	***SE***	***df***	***t* value**	**Pr (>|*t*|)**
*F*1*a*_0_	Intercept	672.18	15.11	85.00	44.49	0.001
	Dental	71.21	15.30	65.00	4.66	0.001
	Labiodental	53.91	15.46	68.00	3.49	0.01
	Velar	119.17	20.22	66.00	5.89	0.001
	AG	−15.45	7.27	2, 143.00	−2.13	0.05
	Voiceless	50.10	10.75	60.00	4.66	0.001
	/i/	−313.32	12.32	60.00	−25.43	0.001
	Velar:AG	−22.52	10.95	7, 025.00	−2.06	0.05
*F*1*a*_1_	Intercept	34.30	3.05	66.00	11.25	0.001
	Dental	−11.05	4.03	66.00	−2.74	0.01
	Labiodental	−8.27	3.85	70.00	−2.15	0.05
	Velar	−11.12	5.33	68.00	−2.09	0.05
	/i/	−27.80	2.36	55.00	−11.76	0.001
*F*1*a*_2_	Intercept	−2.29	0.13	67.00	−17.21	0.001
	Dental	0.64	0.18	78.00	3.54	0.01
	Labiodental	0.40	0.18	99.00	2.27	0.05
	Velar	0.54	0.24	77.00	2.25	0.05
	/i/	1.83	0.10	46.00	18.89	0.001
	Dental:AG	−0.56	0.21	6, 625.00	−2.66	0.01
	Velar:AG	−0.64	0.28	6, 135.00	−2.26	0.05
	Labiodental:Unstressed	−0.63	0.29	97.00	−2.20	0.05
*F*2*a*_0_	Intercept	1, 629.44	37.72	74.00	43.195	0.001
	Labiodental	−116.77	44.51	61.00	−2.624	0.5
	Palatal	209.31	59.77	60.00	3.502	0.001
	AG	−41.38	18.22	3, 043.00	−2.270	0.05
	Unstressed	−119.74	48.13	55.00	−2.488	0.05
	/i/	771.62	28.11	54.00	27.451	0.001
	Labiodental:AG	−63.28	25.37	7, 002.00	−2.494	0.05
	AG:Unstressed	108.10	21.61	6, 977.00	5.003	0.001
*F*2*a*_1_	Intercept	−6.62	6.12	74.00	−1.08	0.283
	Labiodental	18.37	7.32	72.00	2.51	0.05
	Palatal	−20.00	9.29	67.00	−2.15	0.05
	/i/	27.67	5.72	58.00	4.83	0.001
	Dental:AG	12.39	4.93	7, 054.00	2.51	0.05
	Labiodental:AG	16.17	5.50	7, 046.00	2.94	0.01
*F*2*a*_2_	Intercept	−0.06	0.50	64.00	−0.12	0.907
	AG	0.97	0.31	951.00	3.09	0.01
	/i/	−1.97	0.38	53.00	−5.11	0.001
	Dental:AG	−1.04	0.47	7, 046.00	−2.22	0.05
	Labiodental:AG	−1.32	0.48	7, 024.00	−2.73	0.01
	Labiodental:AG:Unstressed	1.64	0.79	6, 983.00	2.07	0.05

As shown from Table 6 Athenian Greek fricatives lowered the *F*1 contour as a whole by an estimate of 15.45 Hz. Also, there were significant effects of the place of articulation, which affected all formant coefficients of *F*1. Most notably, there were significantly effects of the dental, labiodental, and velar fricatives on *F*1. So, *F*1*a*_0_ and *F*1*a*_2_ were found to distinguish Athenian Greek velar fricatives from Cypriot Greek velar fricatives. Also, *F*1*a*_2_ can distinguish Athenian Greek dental fricatives from the Cypriot Greek ones.

A finding that stands out is that the starting frequency of the Athenian Greek *F*2*a*_0_ was overall lower than that of the Cypriot Greek *F*2*a*_0_. *F*2*a*_0_, *F*2*a*_1_, *F*2*a*_2_ can distinguish Athenian Greek and Cypriot Greek fricatives at the labiodental place of articulation. These effects suggest that labiodental fricatives affect the overall shape of *F*2, which results at this place of articulation in distinct formant contours depending on the dialect. Also, the dental place of articulation affects *F*2*a*_1_ and *F*2*a*_2_, which again points to different effects of the dental fricatives on *F*2 in Athenian Greek and Cypriot Greek. Moreover, there were effects of the place of articulation on the formant contour (see the effects of the labiodentals and palatals on *F*2*a*_0_ and *F*2*a*_0_).

*F*3*a*_0_ is overall higher in Athenian Greek than in Cypriot Greek by an estimate of 64 Hz. There were also different effects of the dialect on *F*3*a*_1_ and *F*3*a*_2_, which suggests that the *F*3 contour differs in the two varieties; this finding corroborates earlier studies (e.g., see Themistocleous, 2017b). An important finding is that the place of articulation of fricatives affects the overall shape of the *F*3 contour. Specifically, dental fricatives affect both *F*3*a*_0_ and *F*3*a*_1_. Also, the dialect affected the *F*3*a*_0_ and *F*3*a*_1_ following palatal and velar fricatives.

Athenian Greek and Cypriot Greek palatal and velar fricatives had significantly different effects on the *F*4*a*_0_. Also, the two varieties had different effects on the *F*4*a*_0_ when labiodental fricatives preceded the formant. Overall, these findings are important as they demonstrate that the two dialects have different effects on vowel formants depending on the place of articulation of fricatives that precede the vowel. We did not observe effects of voicing on formant contours, which indicates that the place of articulation has more significant effects on vowel formants than voicing (see also Table 8, 9 for the specific effects of vowel, stress and place of articulation on vowel formants).

**Table 9:** Effects of dialect [Athenian Greek (AG) and Cypriot Greek (CG)], place of articulation, voicing, stress, and vowel on the three polynomial coefficients of *F*3 and *F*4.

		**Estimate**	***SE***	***df***	***t* value**	**Pr (>|*t*|)**
*F*3*a*_0_	Intercept	2, 839.63	30.03	110.00	94.54	0.001
	Dental	−77.62	28.72	74.00	−2.70	0.01
	Labiodental	−195.45	31.57	115.00	−6.19	0.001
	Palatal	433.00	35.47	70.00	12.22	0.001
	Velar	−75.84	35.67	71.00	−2.13	0.05
	AG	64.05	28.32	5, 759.00	2.26	0.05
	/i/	189.31	15.01	54.00	12.61	0.001
	Dental:AG	−82.37	35.23	6, 728.00	−2.34	0.05
	Palatal:AG	−87.26	43.37	6, 833.00	−2.01	0.05
	Velar:Voiceless	−106.93	53.40	109.00	−2.00	0.05
	AG:Voiceless	−61.94	30.47	6, 852.00	−2.03	0.05
*F*3*a*_1_	Intercept	−24.21	6.64	77.00	−3.65	0.001
	Dental	19.77	8.34	67.00	2.37	0.05
	Labiodental	35.60	8.97	93.00	3.97	0.001
	Palatal	−28.39	10.33	64.00	−2.75	0.01
	AG	−17.33	7.00	3, 132.00	−2.48	0.05
	/i/	20.71	4.43	52.00	4.67	0.001
	Dental:AG	23.27	9.19	6, 895.00	2.53	0.05
	Velar:AG	32.37	11.36	6, 897.00	2.85	0.01
	Labiodental:Voiceless	−29.22	12.29	91.00	−2.38	0.05
*F*3*a*_2_	Intercept	1.16	0.53	64.00	2.17	0.05
	AG	1.26	0.37	891.00	3.40	0.01
	/i/	−1.61	0.41	52.00	−3.93	0.001
	AG:Unstressed	−1.38	0.50	6, 989.00	−2.79	0.01
*F*4*a*_0_	Intercept	4, 167.67	47.94	115.00	86.94	0.001
	Dental	−304.74	52.96	82.00	−5.75	0.001
	Labiodental	−378.46	59.33	140.00	−6.38	0.001
	Velar	−239.92	65.60	77.00	−3.66	0.001
	Voiceless	−112.12	48.80	76.00	−2.30	0.05
	/i/	125.45	27.17	55.00	4.62	0.001
	Palatal:AG	−184.67	87.70	6, 859.00	−2.11	0.05
	Velar:AG	−183.96	88.04	6, 761.00	−2.09	0.05
*F*4*a*_1_	Intercept	−40.22	6.89	65.00	−5.84	0.001
	Dental	29.08	9.79	89.00	2.97	0.01
	Labiodental	38.07	9.69	116.00	3.93	0.001
	/i/	13.40	5.22	50.00	2.57	0.05
*F*4*a*_2_	Intercept	2.42	0.55	60.00	4.36	0.001
	Labiodental	−2.01	0.75	88.00	−2.67	0.01
	Labiodental:AG	1.71	0.83	6, 785.00	2.05	0.05

## 4. Study 3: classification study

The preceding sections reported the effects of dialect, place of articulation, stress, and voicing on the temporal and spectral properties of fricatives. Study 3 aims to determine which acoustic properties of fricatives contribute to classification of the dialect (e.g., Athenian Greek and Cypriot Greek). To this purpose, we employed Quinlan's classification algorithm and decision tree, C5.0, using winnowing, a feature selection algorithm that selects features that contribute more to the classification. The predictors included the following:

center of gravity + standard deviation + skewness + kurtosis + duration + F1*a*_0_ + F1*a*_1_ + F1*a*_2_ + F2*a*_0_ + F2*a*_1_ + F2*a*_2_ + F3*a*_0_ + F3*a*_1_ + F3*a*_2_ + F4*a*_0_ + F4*a*_1_ + F4*a*_2_.

To this purpose, the data were separated into a train set consisting of the 90% of the data and an evaluation or test set consisting of the 10% of the data. The analysis was performed with a 10-fold cross-validation repeated 10 times (see for a discussion Ambroise and McLachlan, 2002). The accuracy was used to select the optimal model. The statistical analysis and the classification was carried out in R (R Core Team, 2016). The lme4 R-package, which provided functions for fitting generalized linear mixed models (Bates et al., 2014; Kuznetsova et al., 2016), the caret (Kuhn, 2016), and the C5.0, package (Kuhn et al., 2015) were used for the classification. The final values employed in the selection of the model are reported in the Results section.

### 4.1. Results

Specifically, the model had a high classification accuracy 88% (95% CI[0.85, 0.91], kappa = 0.76). The attribute usage was the following:

(2) $$\begin{array}{c} Classification=100\%\;duration,\;100\%\;centerofgravity,\;100\%\;SD,\\ 100\%\;skewness,\;100\%\;kurtosis,\;100\%\;F1a_{0},\;100\%\;F2a_{0},\\ 100\%\;F2a_{2},\;100\%\;F3a_{0},\;100\%\;F3a_{1},\;100\%\;F4a_{0},\\ 99.89\%\;F4a_{1},\;97.75\%\;F3a_{2},\;96.50\%\;F4a_{2},\;94.34\%\;F1a_{2},\\ 93.98\%\;F1a_{1},\;92.43\%\;F2a_{1}. \end{array}$$

Interestingly, the attribute usage shows that all the spectral moments and the duration contribute greatly to the classification of the dialect resulting in high classification accuracy. In contrast, when we use only the spectral moments or only formant values as predictors the accuracy falls greatly. Specifically, a model with the spectral moments alone resulted into a 83% (95% CI[0.80, 0.86], Kappa = 0.66) classification accuracy, which is almost 5% less accurate than the reported model that employs all measured features whereas the model with the polynomial coefficients only resulted in 66% (95% CI[0.61, 0.70], kappa = 0.3), which is 22% less accurate that the model that employs all features. The comparison of the three classification models suggests that the highest accuracy is achieved only when using all the acoustic properties.

## 5. Discussion

We hypothesized based on perceptual impressionistic evidence that the acoustic structure of Athenian Greek and Cypriot Greek fricatives will differ. To this purpose, we evaluated the information provided by the spectral properties of fricatives and the co-articulatory effects of fricatives on vowel formants. The results demonstrated that the two dialects affect multiple spectral properties of fricatives. These properties are not necessarily different from the ones that distinguish the place of articulation, voicing, and stress. This may come as a striking finding in a tradition of linguistic research that aimed to single out acoustic parameters that associate with a specific phonemic category. Take for example the “locus” theory, which is an approach that hypothesizes that the *F*2 of the vowel is a correlate of fricatives' (and other consonants') place of articulation (see for a discussion Lehiste and Peterson, 1961). A great contribution of this study is that it shows that the “locus” theory underestimates the role of higher order formants, such as *F*3 and *F*4 and that it is not just the *F*2 that conveys information about the place of articulation but all spectral properties of fricatives.

We argue that information about the dialect is encoded by several acoustic features of the fricative spectra. As someone can distinguish a dog from a cat by its picture and/or by the sound it makes, the same is true for speech: a listener can identify the dialect by multiple features that make up fricative spectra and by the effects of fricatives on the adjacent sounds. Specifically, the machine learning and classification algorithm C5.0 employed in this study demonstrated that duration, center of gravity, standard deviation, skewness, kurtosis, and the starting frequency of *F*1*a*_0_, *F*2*a*_0_, *F*3*a*_0_, *F*4*a*_0_, as well as first and second polynomial coefficients of *F*3 and *F*4 play a significant role in the classification of Athenian Greek and Cypriot Greek fricatives. We will come back to this issue later in the discussion, let us however investigate more closely the effects of dialect on fricatives' acoustic features.

One interesting finding is that the *center of gravity* for the labiodental fricative [f], the alveolar fricatives [s] and [z], the voiceless palatal [ç], and the velar fricatives is higher in Cypriot Greek than in Athenian Greek. By contrast, Athenian Greek fricatives [θ] and [ð], [v] and [ʝ] have higher center of gravity than the corresponding Cypriot Greek ones (see section 2.2). These differences were significant and suggest that the center of gravity of fricatives can discriminate the fricative productions of the two varieties. In addition to these effects, Athenian Greek and Cypriot Greek have different effects with respect to stressed vs. unstressed *palatal* fricatives.

Similarly, *standard deviation* varies depending on the dialect. Overall, Cypriot Greek fricatives are characterized by higher standard deviation than Athenian Greek fricatives (e.g., labiodental [v], the dental [ð], the alveolar [s] and [z], the palatals [ç] and [ʝ], and the velars [x] and [ɣ]). This necessarily suggests that Cypriot Greek speakers produce these fricatives with greater variation with respect to the center of gravity than Athenian Greek speakers. By contrast, only the Athenian Greek voiceless labiodental [f] and dental [θ] had higher spectral standard deviation than the corresponding Cypriot Greek fricatives, which suggests that in Cypriot Greek the spectral energy of [f] and [θ] fricative sounds is closer to the center of gravity of these sounds than in Athenian Greek.

Most fricatives are characterized by *positive skewness*; this includes the voiced labiodental, palatal, and velar fricatives. Cypriot Greek fricatives have greater values of skewness than Athenian Greek fricatives. In Cypriot Greek [s] and [z], skewness is negative but positive in Athenian Greek, which suggests that their distribution is left-tailed in Cypriot Greek but right-tailed in Athenian Greek. Another important finding is that *kurtosis* revealed asymmetries in the spectral distribution of Athenian Greek and Cypriot Greek fricatives: voiced fricatives [v ð ʝ ɣ] had high kurtosis whereas the kurtosis for the corresponding voiceless ones was significantly lower. In all cases Cypriot Greek fricatives had higher kurtosis than the Athenian Greek fricatives.

An interesting finding that emerged from Study 1 is that Cypriot Greek *sibilants* [s z] differ from Athenian Greek sibilants in most acoustic properties. First, they associate with higher center of gravity in Hz than the corresponding Athenian Greek sibilants: the center of gravity for the stressed Cypriot Greek [s] was 10,104 Hz whereas the corresponding Athenian Greek [s] was only 6,933 Hz. Similarly, the stressed Cypriot Greek [z] was 8,462 Hz whereas the corresponding Athenian Greek was only 5,718 Hz. Cypriot Greek sibilants had higher standard deviation from the Athenian Greek sibilants. This can be an effect of a different place of articulation of the Cypriot Greek and Athenian Greek sibilant sounds. They also differ in their duration. These findings account for the impressionistic reports from the speakers of these two varieties that [s] and [z] *sound different in Athenian Greek and Cypriot Greek*.

Voiced fricatives are overall shorter than unvoiced fricatives. This finding broadly supports the work of earlier studies showing that *duration* distinguishes voiced and voiceless fricatives: voiceless fricatives are longer than voiced fricatives (Cole and Cooper, 1975; Klatt, 1976; Silbert and de Jong, 2008)^3^. These durational effects are perceptually silent. For example, in a perceptual study of European Portuguese, Pape et al. (2015) showed that there is systematic association of voicing to shorter duration: “The shorter the fricative duration, the more the listeners judged the stimuli as voiced” (Pape et al., 2015, p. 100). Moreover, the place of articulation had significant effects on fricative duration (Silbert and de Jong, 2008; Pape et al., 2015), as each fricative depending on the place of articulation is realized with a different intrinsic duration (Lehiste, 1970; Jongman et al., 2000; Silbert and de Jong, 2008; Iskarous et al., 2011; Pape et al., 2015).

A compelling finding is that Athenian Greek voiceless fricatives are significantly shorter than Cypriot Greek voiceless fricatives. The short Cypriot Greek fricatives, which we measured in this study, are longer that the Athenian Greek fricatives:

$$\begin{array}{c} Athenian\;Greek\;fricatives<Cypriot\;Greek\;short\;fricatives<\\ Cypriot\;Greek\;long\;fricatives. \end{array}$$

Especially, the Athenian Greek alveolar [s] and the palatal [ç] were overall shorter than the corresponding Cypriot Greek ones. The different patterns of duration in Athenian Greek and Cypriot Greek fricatives are captured by the classification model, which ranks the contribution of duration to the classification of dialect higher than all the other features.

These findings might reflect fricative specific duration patterns in the two speech varieties. Evidence from a comparative study of slow and fast productions of Athenian Greek and Cypriot Greek sonorants, that shows that Cypriot Greek singleton sonorants are shorter than Athenian Greek sonorants (Arvaniti, 1999a, 2001), may support this interpretation. Nevertheless, earlier studies on vowels, which show that the Athenian Greek vowels are overall shorter than the corresponding Cypriot Greek vowels (Themistocleous, 2011, 2017a,b), may indicate that the overall Athenian Greek speech is uttered at a faster rate than the Cypriot Greek speech. In any case, further comparative research on the segmental duration of these two varieties is required to establish a proper account of the implications of these findings on fricative duration.

Moreover, there were major progressive coarticulatory effects of fricatives, which affected the starting frequency of *F*1 and its overall shape. *F*1 showed clear effects of voicing, place of articulation, and stress (e.g., see Stevens et al., 1992). This study shows that dialect also affects the *F*1. As was expected, *F*2 interacts with the place of articulation and thus it replicates earlier studies, which show that the place of articulation had significant effects on *F*2, along with voicing and stress (e.g., see Potter et al., 1947; Cooper et al., 1952; Delattre et al., 1955; Stevens and House, 1956; Harris et al., 1958; Lehiste and Peterson, 1961; Öhman, 1966; Fant, 1969; de Manrique and Massone, 1981b; Kewley-Port, 1982; Beckman et al., 2009). However, what this study shows is that the dialect, i.e., Athenian Greek and Cypriot Greek, had significant effects on fricative-vowel coarticulation on *F*2, as well as on *F*3 and *F*4.

So, striking result to emerge from these findings is that the effects of dialect are clearly not isolated on a single acoustic parameter but have manifold effects on fricative spectra. Also, the model suggests that the difference between the fricative productions of a speaker of one dialect from the speaker of another relies on the exact ranking of properties—from more important to less important—and on their interaction. Going back to the point made at the beginning of this section, namely that all measured acoustic properties contribute to the classification of dialect, we need to highlight the contribution of the machine learning and classification model to the understanding of dialectal effects on fricative acoustic structure. The machine learning model is certainly not a cognitive model of how humans perceive and produce fricatives, yet it may shed light on the aspects of the speech signal that are crucial for the classification of dialects and can potentially trigger the attentional mechanisms of speakers and listeners when identifying each dialect. In other words, it can designate which properties listeners may pay attention to when identifying a speaker of a different dialect (even possibly in settings when that speaker code-switches). A future perceptual study should verify these findings from a perceptual point of view.

## 6. Conclusions

The present study was designed to determine the effect of two linguistically proximal varieties of Modern Greek, i.e., Athenian Greek and Cypriot Greek, on the spectral properties of fricatives and on the coarticulatory effects of fricatives on the following vowel. Unlike earlier studies that attempt to single out the invariant acoustic properties of linguistic and sociolinguistic categories in the speech signal, this study reveals a more complex reality where linguistic and sociolinguistic categories influence multiple aspects of the speech signals. A fricative sound depending on the dialect might have higher or lower center of gravity, different degrees of standard deviation, skewness and kurtosis and result on different coarticulatory effects.

## Ethics statement

This study was carried out with written informed consent from all subjects. All subjects gave written informed consent in accordance with the Declaration of Helsinki.

## Author contributions

CT conducted designed and run the experiments, conducted the statistical analysis, and prepared the manuscript.

### Conflict of interest statement

The author declares that the research was conducted in the absence of any commercial or financial relationships that could be construed as a potential conflict of interest.

## Footnotes

^1^In Cypriot Greek there is no phonemic contrast between a long /z:/ and short /z/, so in Table 1 it is unspecified for quantity; however, see for a different approach Arvaniti (1999b) and Armosti (2009) who claim that /z/ is phonemically long, proposing as the default the marked pair of a supposed phonetic contrast.

^2^Cypriot Greek post-alveolar fricatives can be phonemes, especially in loan words or allophones of velar fricatives when the fricative precedes a front vowel. In that case, the post-alveolar fricatives is more marked than the palatal, which is considered more standard (see for example Baltazani et al., 2016, for a current exposition on Modern Greek palatalization).

^3^Note that fricative duration is susceptible to effects of stress and prosodic structure, such as the syllable structure, accentual lengthening, and final lengthening (e.g., de Manrique and Massone, 1981a).
